# Estimation of Weight and Body Measurement Model for Pigs Based on Back Point Cloud Data

**DOI:** 10.3390/ani14071046

**Published:** 2024-03-29

**Authors:** Yao Liu, Jie Zhou, Yifan Bian, Taishan Wang, Hongxiang Xue, Longshen Liu

**Affiliations:** College of Artificial Intelligence, Nanjing Agricultural University, Nanjing 210031, China; 9213020230@stu.njau.edu.cn (Y.L.);

**Keywords:** weight estimation, body measurements, RGB information, multi-head attention, convolutional neural network

## Abstract

**Simple Summary:**

Pig farming plays a critical role in global animal husbandry, with pig weight and body dimensions serving as key indicators of growth and development. Manual measurement methods currently used face challenges like herding difficulties and stress in pigs. To address this, this study introduces a non-contact weight estimation and body measurement model using point cloud data from pig backs. By utilizing a depth camera and advanced algorithms, accurate weight predictions and body size measurements were achieved. The model showed promising results, with reduced errors compared to manual methods, demonstrating its potential for improving efficiency and animal welfare in pig farming practices.

**Abstract:**

Pig farming is a crucial sector in global animal husbandry. The weight and body dimension data of pigs reflect their growth and development status, serving as vital metrics for assessing their progress. Presently, pig weight and body dimensions are predominantly measured manually, which poses challenges such as difficulties in herding, stress responses in pigs, and the control of zoonotic diseases. To address these issues, this study proposes a non-contact weight estimation and body measurement model based on point cloud data from pig backs. A depth camera was installed above a weighbridge to acquire 3D point cloud data from 258 Yorkshire–Landrace crossbred sows. We selected 200 Yorkshire–Landrace sows as the research subjects and applied point cloud filtering and denoising techniques to their three-dimensional point cloud data. Subsequently, a K-means clustering segmentation algorithm was employed to extract the point cloud corresponding to the pigs’ backs. A convolutional neural network with a multi-head attention was established for pig weight prediction and added RGB information as an additional feature. During the data processing process, we also measured the back body size information of the pigs. During the model evaluation, 58 Yorkshire–Landrace sows were specifically selected for experimental assessment. Compared to manual measurements, the weight estimation exhibited an average absolute error of 11.552 kg, average relative error of 4.812%, and root mean square error of 11.181 kg. Specifically, for the MACNN, incorporating RGB information as an additional feature resulted in a decrease of 2.469 kg in the RMSE, a decrease of 0.8% in the MAPE, and a decrease of 1.032 kg in the MAE. Measurements of shoulder width, abdominal width, and hip width yielded corresponding average relative errors of 3.144%, 3.798%, and 3.820%. In conclusion, a convolutional neural network with a multi-head attention was established for pig weight prediction, and incorporating RGB information as an additional feature method demonstrated accuracy and reliability for weight estimation and body dimension measurement.

## 1. Introduction

Pork stands as a primary meat source for humans, and the traditional methods of pig farming can no longer keep pace with the rapidly escalating demand for pork. With the advancement of science and technology, modern techniques are gradually infiltrating various aspects of livestock production [1,2]. Precision livestock farming relies on accurately estimating vital body parameters of livestock, encompassing body size, weight, and physical condition [3]. Currently, manual weighing remains the prevailing method for determining pig weight. However, this approach proves to be time-consuming, labor-intensive, and often induces stress reactions in pigs [4]. The stress experienced by pigs can have detrimental effects on their overall health, leading to issues such as metabolic acidosis, dysfunction of the digestive and respiratory systems, and a compromised immune system. Additionally, if zoonotic diseases emerge, they can swiftly spread among the pig population [5]. These concerns significantly impact pig health, influencing pig selection and breeding, which indirectly affects the quality of pork and subsequently impacts human health.

With the rise of artificial intelligence technology, the livestock and poultry farming industry has entered the era of intelligent farming. Scholar C.P. Schofield [6] utilized image processing techniques to extract and calculate the contour areas of pig images based on grayscale thresholds, discovering a significant correlation between contour area and weight. Similarly, American scholar Grahn [7] integrated the VIA system with the fire system to monitor pig growth rates and provide weight information, but this method requires consistent accuracy during data collection. Estimating the weight of poultry and livestock using 2D images has encountered challenges such as low accuracy and high posture requirements.

In recent years, the use of 3D data for weight estimation in livestock has gained traction. Kongsro [8] employed Kinect depth cameras to capture depth images of pigs, processing these images to establish a linear regression equation for weight prediction. As image processing technology continues to advance, Song et al. [9] achieved success in utilizing machine vision to assess the body condition of cows, capturing images from multiple perspectives and applying techniques like image enhancement and neighborhood averaging, with grading errors remaining within a three-point range and a remarkably low relative error of 0.8%.

In 2013, Liu et al. [10] from China Agricultural University used various techniques to extract 3D coordinate data of pigs, including laser scanners and triangulation. They utilized multiple cameras from various angles to ensure rich 3D data and enhanced estimation accuracy. After processing the initial point cloud data with filtering and denoising, they constructed weight estimation models using different methods, with the RBF neural network model showing the best predictive performance, with an average error of only 1.3%.

Zhang et al. [11] analyzed 3D point cloud data of cows, employing filtering and clustering algorithms to eliminate outliers and segment the cow’s body. Their model exhibited an absolute error within the range of 20–27 kg and a root mean square error of 17 kg. On the other hand, Kwon et al. [12] converted 3D point cloud data and mesh models into 3D voxel grids. While this approach is effective, its limitations hinder widespread practical implementation in farming [13].

In the realm of animal measurement and monitoring, a number of innovative methods have been introduced. Hao H. et al. [14] and Zhang J. et al. [15] put forward enhanced models for pig body size measurement and weight estimation, incorporating advanced techniques such as PointNet++, regression CNN, and deep learning. Furthermore, Meckbach C. et al. [16] and Cang Y. et al. [17] demonstrated promising approaches for precise animal weight monitoring, leveraging convolutional neural networks and intelligent estimation methodologies. Additionally, He H. et al. [18] introduced a novel automatic weight measurement method for pigs based on 3D imaging and a regression network, providing accurate and non-contact weight estimation for pig farming.

This study focuses on processing 3D point cloud data with precision and efficiency, guided by principles of practical production. Voxel filtering and K-means clustering segmentation algorithms were utilized to preprocess the original point cloud, effectively removing noise and segmenting the pig targets. Body dimension features were then extracted, considering their practical utility.

The pig point cloud data were used as input for a deep learning convolutional neural network (CNN) with multi-head attention, creating a weight estimation model. This end-to-end model allows contactless weight prediction based solely on the pig’s back point cloud data. The proposed model offers a streamlined and efficient processing flow, making it directly applicable in the actual production environment of a farm and meeting the demands of practical farming scenarios.

## 2. Materials and Methods

### 2.1. Collection and Processing of Sow Back Video Data

#### 2.1.1. Experimental Environment and Data Collection

**Figure 1:**
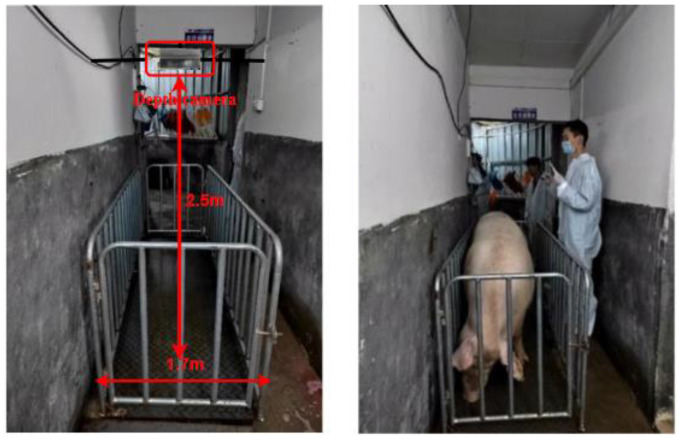
Experimental environment.

**Figure 2:**
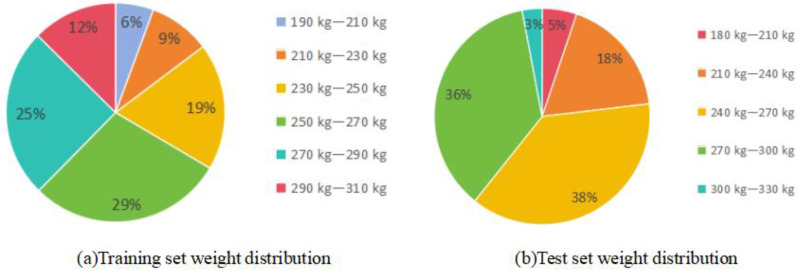
Weight distribution.

**Figure 3:**
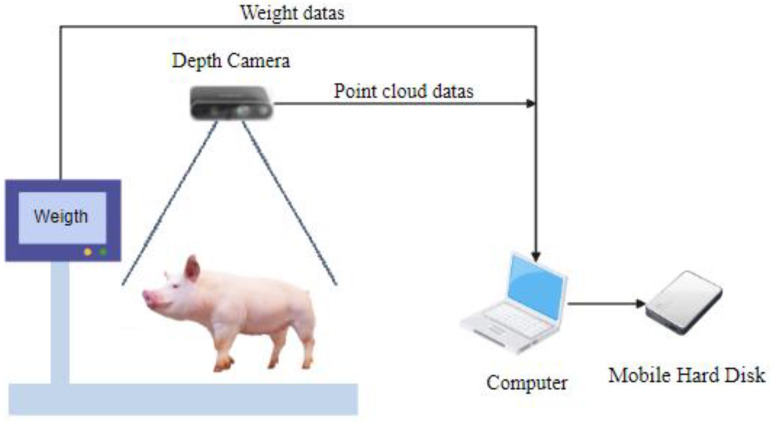
Schematic diagram of the video capture system.

The data in this study were collected at Luo Chen Pig Farm in New Xing County, Yunfu City, Guangdong Province, China. Coordinates: 112.136° E, 22.373° N. The experimental subjects were Yorkshire–Landrace sows (Figure 1). The weight range and proportion of the sows are shown in Figure 2. The location was a pig driving channel with a width of approximately 1.7 m and a height of about 2.5 m. A weighing scale was placed on the side of the wall, and real weight data of the pigs were measured using the scale to provide support for the optimization of the pig weight estimation model. The Inter RealSense D435i depth camera was installed directly above the weighing scale. To ensure the stability of the camera, it was fixed on both sides of the wall using a gantry and a metal hook. The depth camera was connected to a computer, which further connected to a portable hard drive for storing video data, and those are shown in Figure 3. A total of 258 sow videos were collected. To ensure data accuracy, the weighing scale display needed to be zeroed before measurements. The depth camera angle and weighing scale position were calibrated. The camera height was set to 2.5 m to capture the point cloud data from an optimal perspective to ensure comprehensive coverage of the pig’s body.

The Intel RealSense Viewer was used to control the depth camera for recording. Pigs were numbered, and their weights were recorded. After recording a one-minute video, the pigs were herded, and the process was repeated. The fluctuation range of sample pig weights was controlled to enhance the model’s generalization. The data were stored in bag format files on a mobile hard drive.

#### 2.1.2. Data Preprocessing

For point cloud video analysis, an average of 30 frames of 3D point cloud files were parsed per second to optimize the data and improve the accuracy of the weight estimation model. This involved selecting from an extremely large dataset. The initial result was an extensive collection of point cloud files in ply format. These files were then examined using CloudCompare_v2.13.0, and files with issues such as missing point clouds or incomplete pig images were removed. Point cloud files containing complete pig images were retained. To ensure the adequacy of the data and meet the needs of the pig weight estimation model, 50 points cloud files were selected for each of the 200 pigs, which were divided into training and validation sets using the method of random sampling. The validation set accounted for 20% of the total, resulting in a total of 10,000 sets of pig 3D point cloud files. For the remaining 58 pigs, 10 points cloud files were selected for each as the test set, resulting in a total of 580 sets of pig 3D point cloud files.

### 2.2. Processing and Morphometric Measurement of the Point Cloud Data

**Figure 4:**
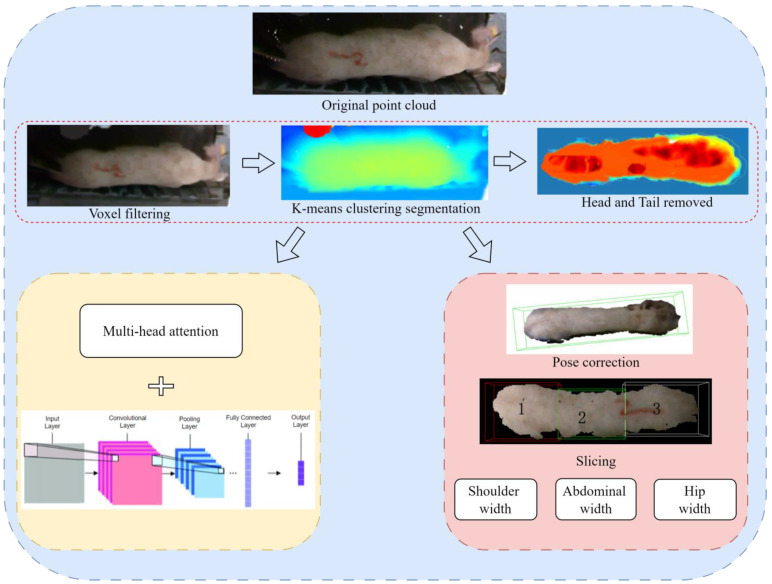
Research flow chart.

This study utilized a depth camera to analyze the 3D image of pigs and applied voxel filtering to filter the back point cloud of the pigs, adjusting the number of points in the back point cloud [19,20]. It utilized the K-means clustering algorithm to segment the point cloud and employed a method based on the local density of 3D point clouds to remove the head and tail of the pig. This study also enveloped the back point cloud of the pig with a minimum bounding box, adjusted the angle to make it parallel to the horizontal line, and extracted the size information of the pig’s back [21]. Finally, it used the position information and RGB information of the back point cloud as input and predicted the weight of the pig through a CNN model with a multi-head attention mechanism. The specific process is shown in Figure 4.

#### 2.2.1. Experimental Setup

**Figure 5:**
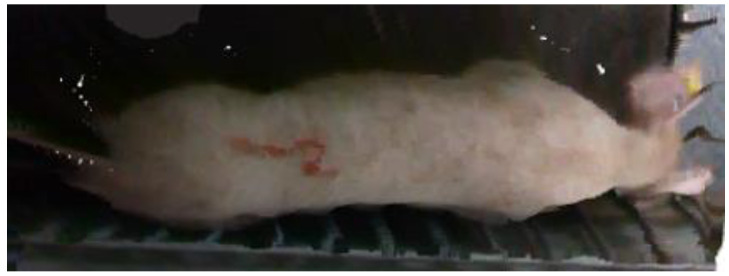
Point cloud display diagram.

The point cloud data processing environment was Python 3.6, PyCharm 2019, and Anaconda under the WIN10 system. Reading the point cloud data and displaying the point cloud file, it is shown in Figure 5.

#### 2.2.2. Voxel Filtering for the Pig Point Cloud Data

**Figure 6:**
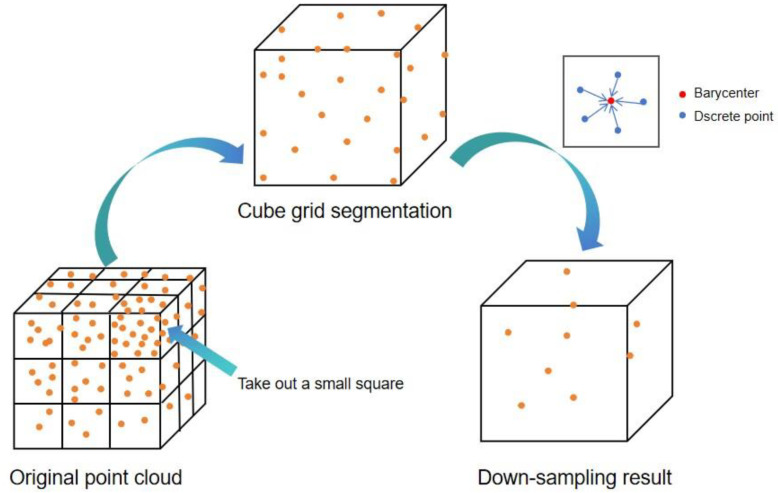
Principle of the voxel filtering operations.

Since the point cloud data is obtained from the surface of pig’s back, the point cloud distribution is relatively stable, and the noise mainly comes from measurement errors during the collection process. The three-dimensional point cloud data are extremely large in volume and irregular in quantity, which has a significant impact on the input of the model [14]. Therefore, in this study, voxel filtering is applied to the point cloud data. Voxel filtering divides the space into equally sized voxel boxes. For each voxel, the surrounding neighboring voxels are averaged with weighted values, and the weights are determined based on the position and distance of the neighboring voxels, reducing the data volume and achieving filtering and down-sampling effects, as shown in Figure 6. Specifically, the 3D space where the point cloud is located is divided into cubic voxel grids with a size of ${\mathrm{edge}}_{\mathrm{length}}$. Each voxel is traversed, and the number of points contained in it is counted. The average value of points in each voxel is calculated as shown in Equation (1).

(1) $$\left\{\begin{array}{c} x_{\mathrm{avg}}=\frac{\left(x_{1}+x_{2}\cdots x_{n}\right)}{n}\\ y_{\mathrm{avg}}=\frac{\left(y_{1}+y_{2}\cdots y_{n}\right)}{n}\\ z_{\mathrm{avg}}=\frac{\left(z_{1}+z_{2}\cdots z_{n}\right)}{n} \end{array}\right.$$

The coordinates $\left(x_{n,}{\;y}_{n},{\;z}_{n}\right)$ represent the coordinate information of each point in the voxel grid.

Replace all the points within the voxel with the calculated average point $\left(x_{\mathrm{avg}},{\;y}_{\mathrm{avg}},{\;z}_{\mathrm{avg}}\right)$. The output of the processed voxel, with the replaced points, can be considered as the filtered point cloud.

The advantage of voxel filtering is its simplicity and ease of use, which can effectively remove outliers and noise from point cloud data. However, it may also result in the loss of details in the point cloud data [22]. Therefore, in this study, experiments were conducted with ${\mathrm{edge}}_{\mathrm{length}}$ of 0.03, 0.04, and 0.05 to evaluate the performance.

#### 2.2.3. Segmentation of Sow 3D Point Cloud Data

The point cloud file underwent point cloud filtering operations, which effectively removed outliers and achieved point cloud denoising. However, after the point cloud filtering, the 3D point cloud image still contained complex environmental backgrounds such as fences and walls. In order to proceed with further research, it was necessary to segment and extract the pig point cloud data from the complex point cloud environment. Therefore, this study will use point cloud segmentation algorithms and analyze the segmentation results to achieve the separation of pigs from complex environments [23].

K-means is an unsupervised learning algorithm that is suitable for point cloud data without label information. It can quickly cluster a large amount of point cloud data [24]. In the pig’s back point cloud data, the background is generally fixed structures such as fences, which have regular shapes. K-means can effectively identify such regular backgrounds and cluster them into one category. The label information from the clustering results can be further utilized to extract the target pig’s point cloud part and achieve segmentation. This provides convenience for subsequent computer vision tasks.

The K-means clustering algorithm randomly selects K points as the centroids, as shown in Equation (2).

(2) $$\mathrm{centroids}=\left\{c1,c2,c3\cdots \mathrm{ck}\right\}$$

where cn represents the initial centroids. Then, the algorithm calculates the distance between each data point and each centroid and assigns the point to the cluster of the centroid with the minimum distance, as shown in Equation (3).

(3) $$\mathrm{distance}\left(\mathrm{point}\;i,\mathrm{centroid}\;j\right)=\sqrt{{\left(\mathrm{point}\;i\;\mathrm{coordinate}-\mathrm{centroid}\;j\;\mathrm{coordinate}\right)}^{2}}$$

where the point i coordinate and centroid j coordinate represent the coordinates of the point and centroid in the point cloud. The algorithm updates the centroids of each cluster by taking the average of all the points in the cluster, as shown in Equation (4).

(4) $$\mathrm{new}\;\mathrm{centroid}\;j=\frac{\sum \mathrm{point}\;\mathrm{in}\;\mathrm{cluster}\;j}{\mathrm{number}\;\mathrm{of}\;\mathrm{points}\;\mathrm{in}\;\mathrm{cluster}\;j}$$

where point in cluster j represents the coordinates of the points in the cluster, and new centroid j represents the updated centroid of the cluster. The algorithm repeats Equations (4) and (5) until the positions of the centroids no longer change.

However, the choice of the number of clusters, K, is difficult. This study will use the elbow method to determine the specific formula, as shown in Equation (5).

(5) $$\mathrm{SSE}=\sum_{i=1}^{k}\sum_{p\in C_{i}}{\left|p-m_{i}\right|}^{2}$$

#### 2.2.4. Removing Head and Tail Based on the Local Density of the 3D Point Cloud

During the data collection process, it was found that the head and tail of the pig exhibit higher levels of activity and have greater uncertainty. Based on previous research, some researchers used convex hull analysis to detect the convexity and concavity of the pig’s contour as a key condition and applied the corner ratio formula proposed by Liu et al. with a threshold of 0.5 to remove the head and tail. However, this method had inherent limitations, as it required manually setting threshold values for the key point extraction, making it difficult to adapt to different datasets. Additionally, convex hull analysis was heavily influenced by obstructed areas and needed to be converted to a 2D contour.

**Figure 7:**
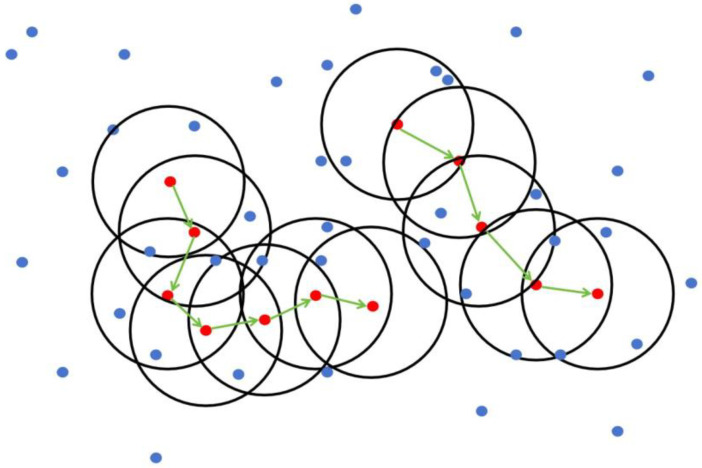
Principle of removing the head and tail.

Based on these issues, this study proposes an improved method for directly removing the head and tail using 3D point clouds, as shown in Figure 7. Specifically:

(1) The original point cloud is divided into equally spaced local blocks using triangulation with a side length of 0.02 m.
(2) The average distance of points within each block is calculated while subtracting the influence of the point with the maximum distance within that block to obtain the weighted local density.
(3) The DBSCAN clustering algorithm is applied to identify the positions of local density maxima. In this case, a fixed neighborhood sample size of 10 and a clustering radius of 0.5 m are used.
(4) The points corresponding to the first and last local density maxima blocks are taken as the initial head and tail endpoints.
(5) Head and tail point clouds are extracted within a range of 0.4 m along the *z*-axis from the endpoints.

This method aimed to differentiate the head and tail regions from the body region based on the spatial density distribution differences, providing an improved approach for removing the head and tail in 3D point cloud data.

#### 2.2.5. Extraction of Body Measurements of a Sow’s Back under a Non-Standard Posture

**Figure 8:**
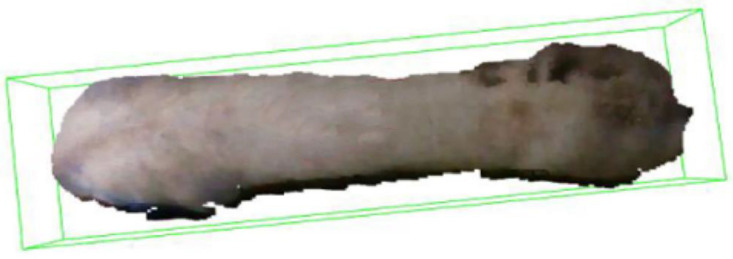
Posture correction effect.

The size of the pig was also a key piece of information for assessing its growth status. In this study, we aimed to utilize the point cloud features of the pig’s back to extract body measurements. However, it was important to note that the posture of the pig’s back during the collection of point cloud data was often inclined, and the posture played a crucial role in extracting body measurements. Ling et al., 2022 [25] divided the posture into standard and non-standard categories and used linear or nonlinear regression methods to build a pig body measurement estimation model for each category. By correcting the body measurement predictions of pigs under non-standard postures, the accuracy of automatic measurements could be significantly improved. However, this study proposed a simpler method to adjust the pig’s horizontal tilt angle using the minimum bounding rectangle so that the back image appeared in a horizontal position, which could also address the posture issue. In this study, we established a minimum bounding box and obtained the pig’s back point cloud OBB (oriented bounding box), as demonstrated in Figure 8, which illustrated the bounding effect.

The OBB encloses the pig’s back point cloud data in the form of a minimum cube. However, the length of the bounding box may have an angular difference with the horizontal direction, which makes it difficult to extract the body measurements of the pig’s back [26]. Therefore, if the angle between the minimum bounding box and the horizontal direction is greater than 1°, the bounding box and the internal point cloud will be rotated accordingly to adjust it to the horizontal direction.

**Figure 9:**
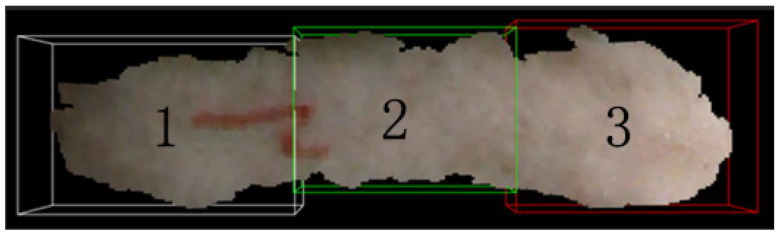
Slicing operation.

After adjusting the posture, we will slice the point cloud in the *x*-axis direction, dividing the pig’s back into three equal parts. Number 3 represents the shoulder, number 2 represents the abdomen, and number 1 represents the hip, as shown in Figure 9.

By dividing the back into three sections in the *x*-axis direction, we can calculate the width of the back at different positions by measuring the length of each section. The formula for calculating the width for any section is shown in Equation (6).

(6) $$L_{\mathrm{ij}}=y_{i\_\max}-y_{i\_\min}$$

where $y_{i\_\max}$ and $y_{i\_\min}$ represent the maximum and minimum values on the *y*-axis at the same *x*-coordinate.

(7) $$L_{j}=\max\left(L_{\mathrm{ij}}\right)$$

where $L_{1}$ represents the shoulder width, $L_{2}$ represents the abdomen width, and $L_{3}$ represents the hip width.

### 2.3. Design of the CNN Model Based on Multi-Head Attention

#### 2.3.1. Model Architecture

In the original CNN model, it consists of 5 convolutional layers, each with an activation function and batch normalization layer [27]. The activation function introduces non-linearity, while batch normalization accelerates training and prevents overfitting. The main function of the convolutional layers is to extract features from the data, with each layer containing a sliding filter for local operations. The number of convolutional layers determines the output channels, the number of feature maps [28]. To extract higher-level features, the number of filters in each convolutional layer is greater than the previous layer. After the last convolutional layer, a max pooling layer is used for down-sampling the feature maps, reducing the parameters and computations while preserving core information. Next is the dropout layer, which randomly drops a portion of neurons to prevent overfitting and improve generalization. Then, a flattening layer is used to convert the multi-dimensional feature maps into a one-dimensional vector for input to the fully connected layers. The fully connected layers are ordinary neural network layers that connect all inputs to outputs, used for regression tasks. This model only contains one fully connected layer, with one output node and a linear activation function, indicating the network is used for regression tasks to predict continuous values. Finally, the stochastic gradient descent (SGD) is used as the optimizer, with the mean squared error as the loss function [29]. The choices of optimizer and loss function affect the updating of network parameters to achieve optimal performance.

**Figure 10:**
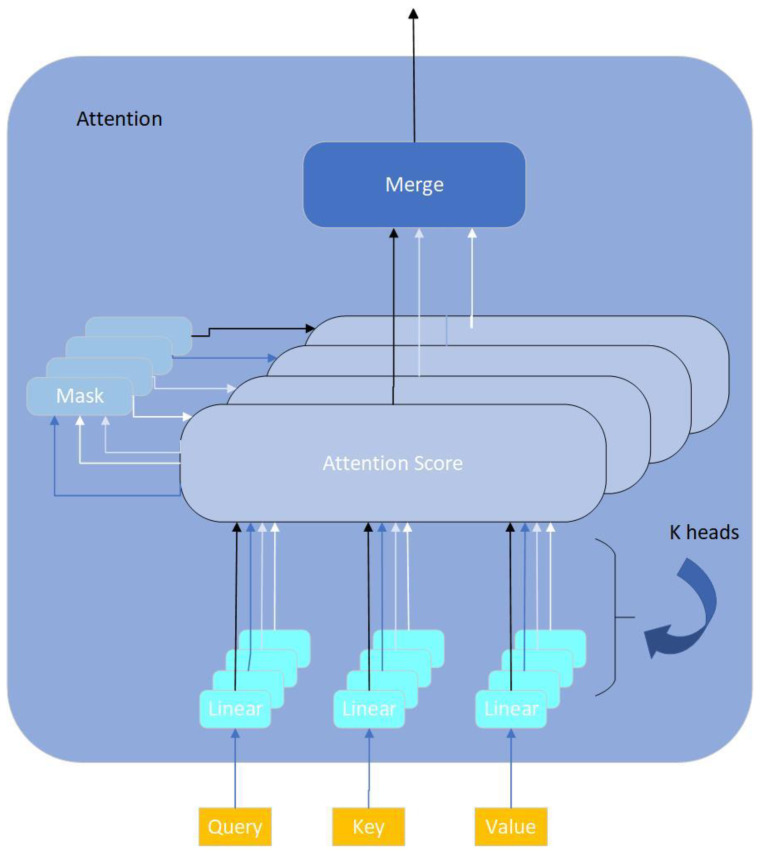
Multi-head attention.

Compared to the original CNN model, we propose a key improvement step where we incorporate a multi-head attention-based point cloud attention mechanism (MACNN) after the CNN convolutional feature extraction. This module includes k parallel attention heads, each consisting of three linear mapping layers, for learning the relationships between points and their neighbors in the point cloud data and enhancing the local feature representation capacity [30]. The attention structure is shown in Figure 10.

This module consists of k parallel attention heads, each of which includes three linear mappings to transform the features. The weight calculation is shown in Equation (8).

(8) $$\mathrm{Attention}\left(Q,K,V\right)=\mathrm{softmax}\left(\frac{{\mathrm{QK}}^{T}}{\sqrt{d}}\right)V$$

In the equation, Q, K, and V represent the query vector, key vector, and value vector, respectively. The softmax function is a normalization function, and d represents the vector dimension.

#### 2.3.2. Model Parameters

The following parameters were used for model training:

The stochastic gradient descent (SGD) algorithm was used for model parameter up-dates. The learning rate was set to 0.001, Considering the large amount of point cloud data, a smaller learning rate was beneficial for convergence. Given the large amount of data in each point cloud sample, the batch size was set to 128. The parameters of the convolutional layers and fully connected layers were initialized using the Xavier uniform distribution. The mean squared error (MSE) was used as the loss function during training and validation. L2 regularization was added to constrain the model complexity, with a regularization strength of λ = 0.001.

These parameter settings ensured a balance between model capacity, training efficiency, and prevention of overfitting.

## 3. Results

### 3.1. Voxel Filtering Results and Analysis

**Figure 11:**
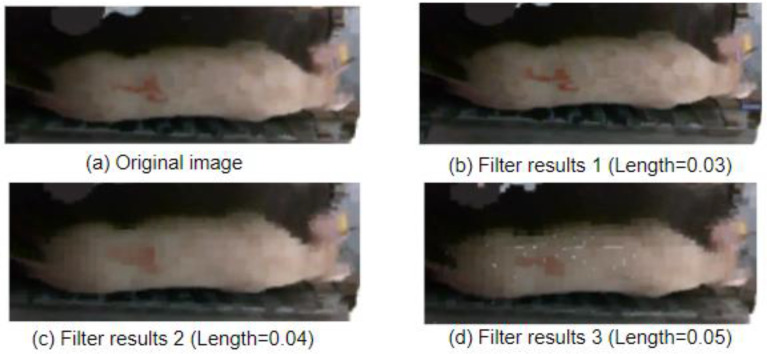
Voxel filtering effect comparison.

The different sizes of cubes correspond to different down-sampling effects, as shown in Figure 11 and in combination with the point cloud quantities in Table 1.

When setting the ${\mathrm{edge}}_{\mathrm{length}}$ to 0.03, for the filtering result, as shown in Figure 11b, most of the point cloud features will not disappear, and the quantity of points will be relatively small. Although, when setting the ${\mathrm{edge}}_{\mathrm{length}}$ to 0.04, for the filtering result, as shown in Figure 11c, a smaller point cloud quantity results in a blur of point cloud features, it is not conducive to extracting point cloud information. When setting the ${\mathrm{edge}}_{\mathrm{length}}$ to 0.05, for the filtering result, as shown in Figure 11d, the point cloud quantity is the minimum, and most of the point cloud features will be missing and the point cloud information will be the least complete. Therefore, in this study, a voxel cube with a side length of 0.03 is chosen for voxel filtering.

### 3.2. Analysis of the K-Means Clustering Results

**Figure 12:**
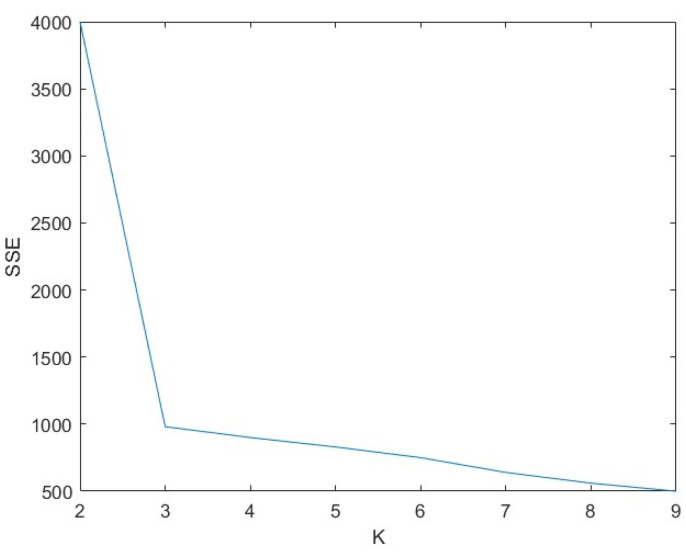
SSE decrease curve.

The value of K is selected based on the sum of squared errors (SSE). However, we do not choose the value of K corresponding to the lowest SSE but rather the value of K where SSE suddenly changes. As shown in Figure 12, K should be selected as 3.

**Figure 13:**
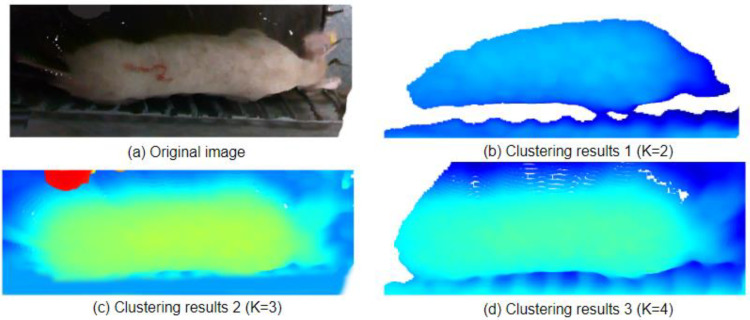
Comparison of the K-means clustering segmentation effects.

Based on the knowledge of the K-means algorithm and the analysis of the clustering results, we selected K values of 2, 3, and 4 to verify the clustering segmentation effect. The comparison of the effects is shown in Figure 13. In Figure 13a, which shows the original image, when we set K as 2, the clustering result in Figure 13b did not separate the main part of the pig, and the number of clusters was relatively small. The edges of the pig’s contour were blurry and not smooth, and some parts of the pig’s point cloud were missing. When we set K as 3, the clustering result in Figure 13c successfully separated the pig completely, with a clear and smooth contour. The head and tail of the pig were separated and included in the pig’s body. When we set the K value as 4, the clustering result in Figure 13d only partially showed the contour of the pig, and the tail of the pig was not separated into a separate cluster, indicating a failed segmentation. Therefore, based on the analysis, when K is set as 3, the segmentation effect is good, successfully extracting the back of the pig.

### 3.3. Analysis of the Removal of the Head and Tail

**Figure 14:**
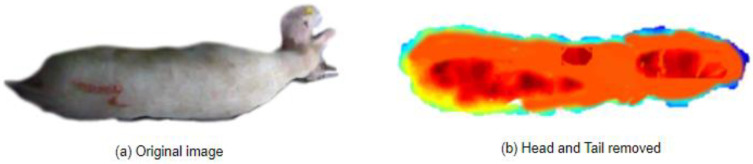
Remove the head and tail results.

The removal results are shown in Figure 14. Figure 14a is the original image, and Figure 14b is the result of using the point cloud local density analysis method for removal. It can be seen that this method effectively filters out noise and adapts to removing the head and tail of different data.

### 3.4. Analysis of the Measurement Results of the Back Point Cloud of Pigs

The comparison between the actual data obtained using a measuring tape and the partial body measurements data obtained from the point cloud data is shown in Table 2. The results indicate that the measurements of shoulder width, abdominal width, and hip width obtained from the point cloud data have a higher accuracy. However, further research is needed for these parameters, mainly due to variations in the posture of the pigs. It is also important to consider that there may be errors in the measurements taken with a measuring tape, which would require two people to work together and may introduce subjective errors during positioning and data reading. Although this study corrects the pig’s posture using the minimum bounding rectangle method, it can only reduce some of the errors but cannot completely eliminate them [22] (Table 3).

### 3.5. Analysis of the Results of the Model

#### 3.5.1. Feature Extraction

The point cloud data contain three-dimensional coordinate information and point cloud RGB information. The former can reflect the characteristics of objects such as size and structural shape, while the latter can reflect surface texture and material features. These features are closely related to the weight of pigs. By using neural networks for learning and extraction, it is possible to discover advanced features hidden in the point cloud data, such as local shapes, and enhance the representation capability of these features. However, theoretically, the point cloud RGB information itself cannot directly support weight prediction. But within the framework of neural network learning, it can be used as an additional feature, which may contribute to improving overall prediction performance.

**Figure 15:**
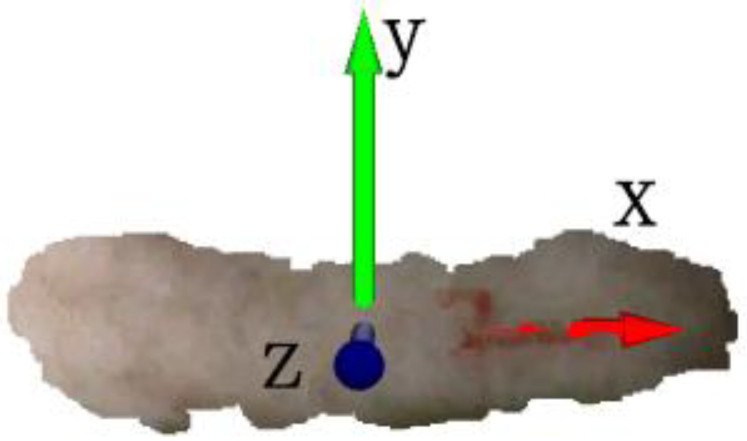
Three-dimensional coordinate system.

Therefore, this study will create two types of datasets: one with three-dimensional coordinate information and the other with both three-dimensional coordinate and point cloud RGB information. The coordinate origin will be set at the center of the pig point cloud dataset, establishing a three-dimensional coordinate system, as shown in Figure 15.

The partial point cloud datasets for each pig are shown in Table 4 and Table 5.

Finally, the feature vectors are added to the list as model inputs. The data are divided into training and validation sets, with the validation set accounting for 20%.

#### 3.5.2. Analysis of the Results of the Pig Weight Estimation Model

**Figure 16:**
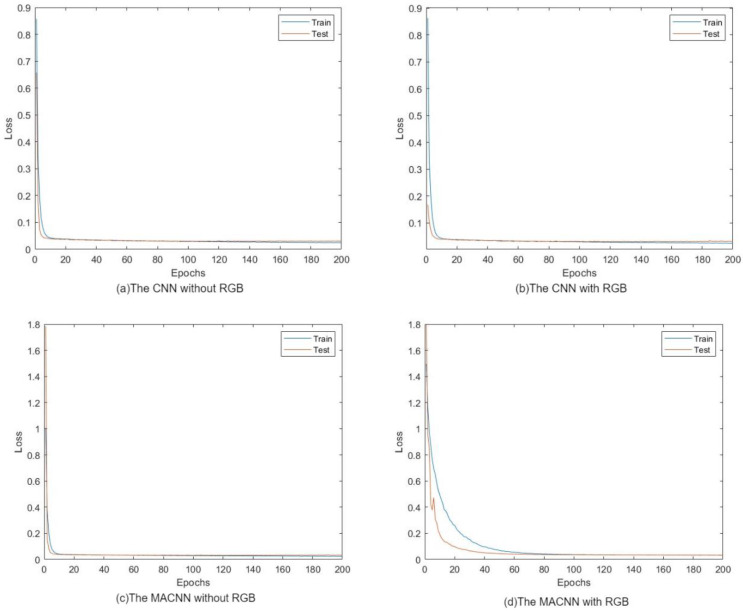
Loss diagram.

From the loss curve during training (Figure 16), it can be seen that the loss function of the training set continues to decrease. The convolutional neural network (CNN) based on the multi-head attention mechanism effectively trains the data. The selected parameters are appropriate, and the model converges quickly during subsequent iterations. The loss function in the test set also continues to decrease and reaches the same value as the training set. This indicates that the test results are consistent with the training results and the model has good generalization ability.

**Figure 17:**
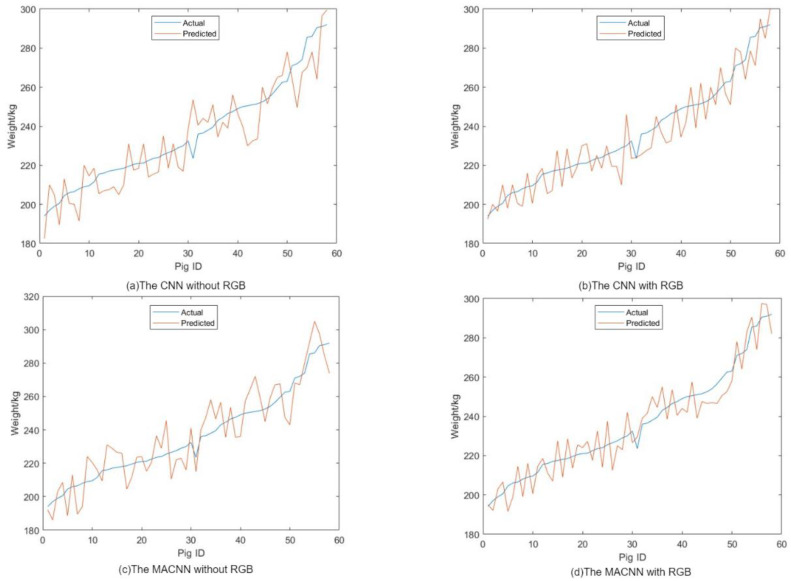
Weight estimation.

Selecting 58 pigs as a test set, the performance of four models was evaluated. The line graph in Figure 17 shows the comparison between the true and estimated values of pig weights. It can be observed that the results obtained from the CNN incorporating RGB information and the multi-head attention mechanism are closer to the actual values.

To validate the accuracy of the pig weight estimation model, the true values of pig weights were compared with the estimated values, and three evaluation metrics were calculated: mean absolute error (MAE), mean absolute percentage error (MAPE), and root mean square error (RMSE). The error values between the true and estimated values for each pig are presented in the comparison table.

Table 6 compares the weight prediction performance of CNN models without RGB information and CNN models with RGB information on the validation set. The CNN model incorporating RGB information as an additional feature demonstrates better performance in all three evaluation metrics: the RMSE decreased by 1.792 kg, the MAPE decreased by 0.47%, and the MAE decreased by 1.505 kg. This indicates that incorporating RGB information as an additional feature can improve the prediction accuracy of the CNN model.

Similarly, Table 7 presents the comparison of MACNN models without RGB information and MACNN models with RGB information on the validation set. Like the CNN models, the MACNN model incorporating RGB information as an additional feature outperforms the MACNN model without RGB information in all metrics: the RMSE decreased by 2.469 kg, the MAPE decreased by 0.8%, and the MAE decreased by 1.032 kg. This further suggests that incorporating RGB information as an additional feature representation can enhance the pig weight prediction capability of the multi-head attention neural network model.

Overall, both CNN and MACNN models show improved weight prediction performance on the validation set when RGB information is included in the feature representation. This indicates that utilizing the full information from the point cloud can enhance the models’ ability to learn implicit relationships and improve their application effectiveness.

### 3.6. Analysis of the Process Automation Results

#### 3.6.1. Building a GUI

**Figure 18:**
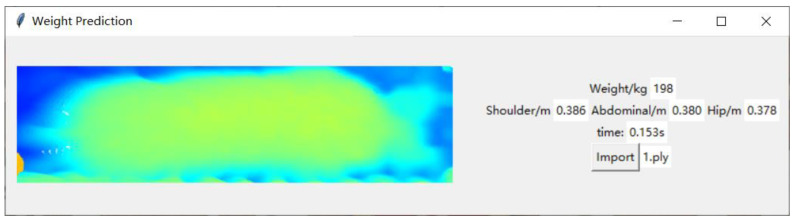
GUI.

Since this research has practical application scenarios that require pen-separated feeding for pigs of different weight ranges, this study designed a GUI application program using Python to achieve end-to-end automation. This program only needs to import the point cloud file, and it can automatically measure the weight and body dimensions, display the prediction time, etc., as shown in Figure 18.

#### 3.6.2. Analysis of the Model Prediction Time

This research is mainly applied to the task of pen-separated feeding for pigs and needs to be installed on the pig driving aisle. If the time is too long, it will cause stress reactions in the pigs, so there is a high requirement for timeliness. Tests were conducted on the built interface, and 1, 10, and 20 pigs were randomly sampled for testing. The prediction times obtained are shown in Table 8, with an average prediction time of 0.151 s, indicating high timeliness.

## 4. Discussion

This study aims to develop a pig weight estimation model using 3D point cloud data captured from pigs. The objective is to achieve accurate and efficient weight estimation without the need for physical contact, thereby minimizing the stress response experienced during manual weight measurement and reducing the risk of zoonotic diseases associated with human–pig contact. The model presented in this study offers several characteristics:

1. Contactless measurement: Unlike traditional contact-based methods, this study employs a non-contact approach for measurement [31]. By eliminating physical contact, this method reduces stress in pigs and minimizes the frequency of human–pig interaction, thereby lowering the risk of zoonotic diseases.

2. Weight estimation and body dimension measurement based on 3D point cloud data: Utilizing depth cameras, this study captures 3D point cloud data of the pig’s back. Through analysis and interpretation of this data, the estimation of pig weight is accomplished. In contrast to the 2D poultry weight estimation method proposed by Dohmen et al. [32], the developed model in this study provides additional information such as hip width, shoulder width, and abdominal width.

3. Before the model training, we adopted a manual data processing approach to ensure the accuracy of the model. Subsequently, we have created an automated data processing workflow using a Python GUI, including data cleaning and preprocessing steps. These tools can automatically execute data processing tasks based on predefined rules and conditions, enhancing efficiency and reducing manual intervention.

4. The pig weight prediction method proposed in this study based on point cloud information differs from existing research in several aspects. Compared to the method of Zhang J. et al. [15]. that used CNN regression to analyze body size parameters, this study directly input pig back point cloud information (such as coordinates) into a CNN for weight prediction while incorporating a multi-head attention mechanism to improve model accuracy. Unlike the research by Meckbach C. et al. [16] that used two-dimensional and depth images as input, this study conducted preprocessing such as background segmentation and denoising in the depth image processing, significantly improving the model’s running speed. In contrast to the direct use of depth images as input by Cang Y. et al. [17], this study extracted feature information such as coordinates from depth images as input to the network, achieving not only weight prediction but also pig body size measurement. Compared to the network structure with dual-branch convolution and a multi-head self-attention mechanism used by He H. et al. [18], this study also introduced a multi-head attention mechanism, significantly improving the effectiveness of the CNN in processing input point cloud information and demonstrating an advantage in the model running speed. Overall, this study has made innovative improvements in data preprocessing, network structure design, and model performance, providing new ideas for machine vision-based automatic pig weight estimation.

5. By incorporating the RGB information as additional feature representation into the MACNN, the model’s performance on the validation set has improved. Specifically, integrating the RGB information as an extra feature resulted in a decrease of 2.469 kg in the RMSE, a 0.8% reduction in the MAPE, and a 1.032 kg decrease in the MAE for the MACNN model. This indicates that the RGB information contributes to better weight prediction in pigs by the MACNN model. The RGB information provides additional features that enhance the model’s predictive ability, thereby improving the model’s performance.

6. The model has limitations, such as its relatively low level of automation, as it still requires some degree of manual intervention. Additionally, it may not be well suited for cup-shaped or angular objects, which could introduce significant errors. Moreover, significant errors may also occur when dealing with objects with high curvature or excessively large original feature elements. Reconstructing point clouds from the pig’s legs and head poses more challenges, and although these body parts contribute less to the overall weight, they still impact the model’s accuracy.

## 5. Conclusions

In this study, we propose a method for estimating the weight and body size of sows based on 3D point cloud data. We capture point cloud data of the pig’s back using a depth camera and preprocess the point cloud using voxel filtering and the K-means clustering segmentation algorithm to effectively extract the pig’s back. By adjusting the posture using the minimum bounding rectangle, we extract the body size information of the pig, such as shoulder width, abdominal width, and hip width. We establish a weight prediction model based on point cloud data using a CNN with a multi-head attention, incorporating RGB information as an additional feature.

We conducted experiments on RGB information and created two datasets: one with three-dimensional coordinate information and the other with both three-dimensional coordinates and point cloud RGB information. The experimental results showed that incorporating RGB information as an additional feature improved the weight prediction performance of the CNN and MACNN on the validation set compared to using only three-dimensional coordinate information.

Specifically, for the CNN, incorporating RGB information as an additional feature resulted in a decrease of 1.792 kg in the RMSE, a decrease of 0.47% in the MAPE, and a decrease of 1.505 kg in the MAE. The model’s performance improved with the addition of the RGB information. For the MACNN, incorporating RGB information as an additional feature resulted in a decrease of 2.469 kg in the RMSE, a decrease of 0.8% in the MAPE, and a decrease of 1.032 kg in the MAE. This also indicates that incorporating RGB information as an additional feature representation enhances the ability of the multi-head attention neural network model to predict pig weights.

The model was tested on 58 sows. The results showed that the average absolute error of weight estimation was 11.552 kg, the average relative error was 4.812%, and the root mean square error was 11.181 kg. In addition, we measured the shoulder width, abdominal width, and hip width and compared them with manual measurements. The results showed average relative errors of 3.144%, 3.798%, and 3.820%, respectively.

Compared with traditional manual measurement methods, this model achieves contactless and efficient weight monitoring, providing reliable technical support for pig farming. Overall, this method performs well in terms of simplicity, prediction accuracy, and practicality. However, we acknowledge that this method may not be suitable for cup-shaped or angular objects. In addition, a larger curvature of the target object or excessively large original feature elements may introduce larger errors. Further exploration is needed in future research to address these issues.

We plan to continue acquiring more point cloud data of sows in the existing environment to further improve the accuracy of weight and body size prediction. It is worth noting that reconstructing the point cloud of the sow’s legs and head is more challenging. Although these body parts contribute less to the overall weight, they still affect the accuracy of the model. Therefore, in future point cloud processing, the focus will focus on how to effectively utilize these parts to improve the accuracy of the model.

## Figures and Tables

**Table 1:** Comparison table of voxel down-sampling.

$\mathrm{Voxel}\;\mathrm{Cube}\;Edge_{length}$	Original Point Cloud	Down-Sampled Point Cloud Count
0.03	236,391	18,715
0.04	236,391	11,391
0.05	236,391	7579

**Table 2:** Body measurement comparison.

Number	True Shoulder	Extraction Shoulder	True Abdominal	Extraction Abdominal	True Hip	Extraction Hip
1	0.395	0.386	0.481	0.492	0.404	0.414
2	0.433	0.451	0.368	0.357	0.378	0.365
3	0.291	0.285	0.324	0.304	0.321	0.306
4	0.411	0.396	0.316	0.325	0.422	0.434
5	0.491	0.474	0.395	0.417	0.335	0.323
6	0.415	0.425	0.361	0.373	0.381	0.362
7	0.401	0.385	0.362	0.350	0.401	0.420

**Table 3:** Measurement error.

Shoulder Error	Abdominal Error	Hip Error
3.144%	3.798%	3.820%

**Table 4:** Not including RGB information.

x	y	z
−0.26387	0.02476	−1.68700
−0.25939	0.02476	−1.68700
−0.25431	0.02470	−1.68300
−0.24984	0.02470	−1.68300
−0.24537	0.02470	−1.68300
−0.24148	0.02476	−1.68700
−0.23770	0.02483	−1.69200
−0.23321	0.02483	−1.69200

**Table 5:** Including RGB information.

x	y	z	Red	Green	Blue
−0.26387	0.02476	−1.68700	137	128	119
−0.25939	0.02476	−1.68700	137	130	120
−0.25431	0.02470	−1.68300	136	127	122
−0.24984	0.02470	−1.68300	134	119	118
−0.24537	0.02470	−1.68300	125	110	109
−0.24148	0.02476	−1.68700	123	108	107
−0.23770	0.02483	−1.69200	125	111	105
−0.23321	0.02483	−1.69200	128	109	103

**Table 6:** The error of the CNN with or without RGB information.

The CNN without RGB Information			The CNN with RGB Information		
RMSE	MAPE	MAE	RMSE	MAPE	MAE
14.683 kg	5.80%	14.188 kg	12.891 kg	5.33%	12.683 kg

**Table 7:** The error of the MACNN with or without RGB information.

The MACNN without RGB Information			The MACNN with RGB Information		
RMSE	MAPE	MAE	RMSE	MAPE	MAE
14.021 kg	5.61%	12.213 kg	11.552 kg	4.81%	11.181 kg

**Table 8:** Selecting samples of different sizes and conducting statistical analysis for predicting the time.

Number	1	10	20
Prediction time/s	0.153	1.482	1.511

## Data Availability

Data are contained within the article.

## References

[1] Chen C., Zhu W., Steibel J., Siegford J., Han J., Norton T. (2020). Recognition of feeding behaviour of pigs and determination of feeding time of each pig by a video-based deep learning method. Comput. Electron. Agric..

[2] Delsart M., Pol F., Dufour B., Rose N., Fablet C. (2020). Pig farming in alternative systems: Strengths and challenges in terms of animal welfare, biosecurity, animal health and pork safety. Agriculture.

[3] Silva S.R., Araujo J.P., Guedes C., Silva F., Almeida M., Cerqueira J.L. (2021). Precision technologies to address dairy cattle welfare: Focus on lameness, mastitis and body condition. Animals.

[4] Rosanowski S.M., Magouras I., Ho W.C., Yiu WC J., Pfeiffer D.U., Zeeh F. (2023). The challenges of pig farming in Hong Kong: A study of farmers’ perceptions and attitudes towards a pig health and production management service. BMC Vet. Res..

[5] Rajić A., Chow E.Y.W., Wu J.T.Y., Deckert A.E., Reid-Smith R., Manninen K., Dewey C.E., Fleury M., McEwen S.A. (2007). Salmonella infections in ninety alberta swine finishing farms: Serological prevalence, correlation between culture and serology, and risk factors for infection. Foodborne Pathog. Dis..

[6] Schofield C.P. (1990). Evaluation of image analysis as a means of estimating the weight of pigs. J. Agric. Eng. Res..

[7] Grahn R.A., Biller D.S., Young A.E., Roe B.A., Qin B., Lyons L.A. (2004). Genetic testing for feline polycystic kidney disease. Anim. Genet..

[8] Kongsro J. (2014). Estimation of pig weight using a Microsoft Kinect prototype imaging system. Comput. Electron. Agric..

[9] Song X., Bokkers E.A.M., van Mourik S., Groot Koerkamp P.W.G., van der Tol P.P.J. (2019). Automated body condition scoring of dairy cows using 3-dimensional feature extraction from multiple body regions. J. Dairy Sci..

[10] Liu T., Li Z., Teng G., Luo C. (2013). Prediction of pig weight based on radical basis function neural network. Nongye Jixie Xuebao/Trans. Chin. Soc. Agric. Mach..

[11] Zhang Z., Zhou H., Wang S., Lv Y., Zheng X., Zeng L. (2022). Research on global actual measurement of indoor surface flatness and verticality Based on sparse point cloud. J. Phys. Conf. Ser..

[12] Kwon K., Mun D. (2022). Iterative offset-based method for reconstructing a mesh model from the point cloud of a pig. Comput. Electron. Agric..

[13] Hegde S., Gangisetty S. (2021). PIG-Net: Inception based Deep Learning Architecture for 3D Point Cloud Segmentation. arXiv.

[14] Hao H., Jincheng Y., Ling Y., Gengyuan C., Sumin Z., Huan Z. (2023). An improved PointNet++ point cloud segmentation model applied to automatic measurement method of pig body size. Comput. Electron. Agric..

[15] Zhang J., Zhuang Y., Ji H., Teng G. (2021). Pig weight and body size estimation using a multiple output regression convolutional neural network: A fast and fully automatic method. Sensors.

[16] Meckbach C., Tiesmeyer V., Traulsen I. (2021). A promising approach towards precise animal weight monitoring using convolutional neural networks. Comput. Electron. Agric..

[17] Cang Y., He H., Qiao Y. (2019). An intelligent pig weights estimate method based on deep learning in sow stall environments. IEEE Access.

[18] He H., Qiao Y., Li X., Chen C., Zhang X. (2021). Automatic weight measurement of pigs based on 3D images and regression network. Comput. Electron. Agric..

[19] He T., Hong L., Kaufman A., Varshney A., Wang S. Voxel-based object simplification. Proceedings of the IEEE Visualization Conference.

[20] Meng H.Y., Gao L., Lai Y.K., Manocha D. VV-net: Voxel VAE net with group convolutions for point cloud segmentation. Proceedings of the IEEE International Conference on Computer Vision.

[21] Ikotun A.M., Ezugwu A.E., Abualigah L., Abuhaija B., Heming J. (2023). K-means clustering algorithms: A comprehensive review, variants analysis, and advances in the era of big data. Inf. Sci..

[22] Li G., Liu X., Ma Y., Wang B., Zheng L., Wang M. (2022). Body size measurement and live body weight estimation for pigs based on back surface point clouds. Biosyst. Eng..

[23] Biasutti P., Lepetit V., Aujol J.F., Bredif M., Bugeau A. LU-net: An efficient network for 3D LiDAR point cloud semantic segmentation based on end-to-end-learned 3D features and U-net. Proceedings of the 2019 International Conference on Computer Vision Workshop, ICCVW.

[24] Ahmed M., Seraj R., Islam S.M.S. (2020). The k-means algorithm: A comprehensive survey and performance evaluation. Electronics.

[25] Ling Y., Jimin Z., Caixing L., Xuhong T., Sumin Z. (2022). Point cloud-based pig body size measurement featured by standard and non-standard postures. Comput. Electron. Agric..

[26] Chang J.W., Wang W., Kim M.S. (2010). Efficient collision detection using a dual OBB-sphere bounding volume hierarchy. CAD Comput. Aided Des..

[27] Sakib S., Ahmed N., Kabir A.J., Ahmed H. (2019). An Overview of Convolutional Neural Network: Its Architecture and Applications. Preprints.

[28] Kermanidis K.L., Maragoudakis M., Krichen M. (2023). Convolutional Neural Networks: A Survey. Computers.

[29] Gower R.M., Loizou N., Qian X., Sailanbayev A., Shulgin E., Richtárik P. SGD: General Analysis and Improved Rates. Proceedings of the 36th International Conference on Machine Learning.

[30] Nebili B., Khellal A., Nemra A., Mascarilla L. (2023). Augmented Convolutional Neural Network Models with Relative Multi-Head Attention for Target Recognition in Infrared Images. Unmanned Syst..

[31] Li X., Yu M., Xu D., Zhao S., Tan H., Liu X. (2023). Non-Contact Measurement of Pregnant Sows’ Backfat Thickness Based on a Hybrid CNN-ViT Model. Agriculture.

[32] Dohmen R., Catal C., Liu Q. (2022). Computer vision-based weight estimation of livestock: A systematic literature review. N. Z. J. Agric. Res..

